# Health Disparity between the Older Rural-to-Urban Migrant Workers and Their Rural Counterparts in China

**DOI:** 10.3390/ijerph17030955

**Published:** 2020-02-04

**Authors:** Dan Li, Zhongliang Zhou, Chi Shen, Jian Zhang, Wei Yang, Rashed Nawaz

**Affiliations:** 1 School of Public Policy and Administration, Xi’an Jiaotong University, Xi’an 710049, China; dandanli@stu.xjtu.edu.cn (D.L.); shenchi@stu.xjtu.edu.cn (C.S.); zhjian.2004@stu.xjtu.edu.cn (J.Z.); nawazrashed@stu.xjtu.edu.cn (R.N.); 2 Health Science Center, Xi’an Jiaotong University, Xi’an 710061, China; sxxayw@stu.xjtu.edu.cn

**Keywords:** health disparity, older rural-to-urban migrant workers, older rural dwellers, CEM

## Abstract

*Background:* China’s older rural-to-urban migrant workers (age 50 and above) are growing old, but comparative health research on older rural-to-urban migrants in China is still in its infancy. The aim is to explore the health status of older rural-to-urban migrant workers in China; as well as to identify health disparity between older rural-to-urban migrant workers and older rural dwellers. *Methods:* This study employed self-assessed health status (SAH) and chronic disease condition to explore the health status. Coarsened exact matching (CEM) was employed to improve estimation of causal effects. Fairlie’s decomposition analysis was conducted to find the health disparity. *Results:* Older rural-to-urban migrant workers were more prone to suffer from chronic diseases, but they had higher SAH when comparing older rural dwellers. Fairlie’s decomposition analysis indicated 10.44% of SAH disparities between two older groups can be traced to bath facility; 31.34% of chronic diseases disparities can be traced to educational attainment, sleeping time and medical scheme. *Conclusions:* This is the first comparative study examining health disparity focusing on older rural-to-urban migrant workers. Our study highlighted substantial health disparities between older rural-to-urban migrant workers and their older rural dwellers. Based on the contributing factors, government should take the drivers of health disparities into consideration in policy setting.

## 1. Background

With the repaid urbanization and industrialization since the 90s of last century, the number of rural-to-urban migrant workers had experienced a dramatic raise. With the passage of time, a number of rural-to-urban migrant workers have entered the old age and are developing a special kind of labor group, namely older rural-to-urban migrant workers (age 50 and above) [1,2,3]. Under the dual influence of aging population and economic development in China, older rural-to-urban migrant workers have become a constant social phenomenon. According to the National Bureau of Statistics (NBC) [1], older rural-to-urban migrant workers accounted for 17.9% of rural-to-urban migrant workers in 2015, and older rural-to-urban migrant workers increased by 3.6% from the period of 2011–2015. Older rural-to-urban migrant workers have made substantial contribution to cities; however, due to the Chinese household registration system (Hukou), they tend to be “sojourners”: they maintained temporary and circular patterns of movement between cities and their home villages [4]. Older rural-to-urban migrant workers’ health vulnerability may lead to delay or failure to report health concerns or receive treatment, which can cause more serious public health problems. Notably, the recognition of their health status is a crucial topic that we cannot avoid. 

There are mixed results of the health effects on migration. Extensive literature studies found that rural-to-urban migrant workers are healthier than their counterparts in receiving countries or their non-migrating peers at home because of medical pre-screening, selection bias and healthy behaviors, referred as the “healthy migrant effect” [5,6,7]. Chen et al. re-examined the healthy migrant phenomenon in China and found that rural-to-urban migrant workers had better physical health status than their non-migrant counterparts. In contrast, several studies [8,9] had demonstrated that rural-to-urban migrant workers were more susceptible to poor health outcome. Poulter et al. stated that migrants in cities in Kenya often suffered from hypertension and had higher blood pressure than non-migrants. Li et al. discovered that rural-to-urban migrant workers in Beijing City suffered from poorer mental health status than their counterparts in the rural areas. 

Many studies [10,11,12] have investigated the health of “floating elderly” in China whose original intention is grand-children’s care, housekeeping, better health care services and family care—different from that of older rural-to-urban migrant workers. Few studies have been conducted to focus on older Chinese rural-to-urban migrant workers. To our best of knowledge, only four empirical studies in China have specially focused on the health of older rural-to-urban migrant workers in China. Hong et al. [13] examined health inequalities of older rural-to-urban migrant workers (aged 45–70) in three cities of China and found that the richer were at a disadvantage in health disparities. Wu et al. [2] compared older rural-to-urban migrant workers (aged 50 and above) with the middle-aged rural-to-urban migrant workers (aged 30–49) and young rural-to-urban migrant workers (aged 16–29), and reported that older rural-to-urban migrant workers’ mental health was inferior to that of middle-aged rural-to-urban migrant workers and young rural-to-urban migrant workers. However, Hong and Wu did not compare the health status with rural counterparts. Tong et al. [14] analyzed the relationship between age (15–60 years old) and health across different migration status groups, and found that rural ever-migrants reported worse health than urban non-migrants, especially at older ages. Zhu et al. [15] described the health differences between the older rural-to-urban migrant workers (aged 60 and above) and other rural older and young rural-to-urban migrant workers. However, Tong and Zhu did not illuminate the reasons for the health disparities.

Therefore, this research aimed to decompose disparities in health outcomes between older rural-to-urban migrant workers in China and their rural counterparts into its contributory factors. Furthermore, to guarantee better balance of empirical distributions of the covariates between the comparison groups [16,17], we commenced coarsened exact matching (CEM) to compare health disparities by matching older rural-to-urban migrant workers and older rural dwellers. This study may contribute to the literature on older rural-to-urban migrant workers in China and more importantly, the results also had important implications for the health disparity of rural older groups, urging the government to take full account of heterogeneity in the formulation of health policies and health interventions.

## 2. Methods

### 2.1. Data

In the current study, we used a nationally representative cross-sectional data from the Chinese Health and Retirement Longitudinal Study (CHARLS) conducted in 2015. CHARLS is conducted every two years., and CHARLS attempts to set up a high quality public micro-database, which could provide a wide range of information from socio-economic status to health conditions, to serve the needs of scientific research on Chinese population aged 45 and above [18]. To ensure sample representativeness, respondents were chosen randomly by PPS (probability proportional to size) in 450 village-level units (villages in rural areas and urban communities in urban areas), 150 county-level units and 28 provinces. Within each household, one person aged 45 and older was randomly chosen to be the main respondent and their spouse was automatically included. CHARLS in 2015 successfully interviewed about 23,000 individuals in about 12,400 households, reflecting the Chinese mid-aged and elderly population collectively. This data-set takes account of detailed demographic, health, economic and cognitive information on individuals who are part of this study. Detailed description of the sampling method, quality assurance measures and the questionnaire has been previously published [18]. All data will be available for inspection one year after the end of data collection [19].

In this study, we restricted our analyses to respondents aged 50–65, in order to exclude who had already exited labor force. Older rural-to-urban migrant workers are defined as, those who are permanently based in city, aged 50–65, with rural Hukou and are employed for more than six months. Older rural dwellers are those defined as who are permanently based in village, aged 50–65, with rural Hukou and are not employed, or employed for less than 6 months.

### 2.2. Health Status Measurement

The self-assessed of health status (SAH) and chronic disease condition are important metrics that can be utilized to reflect health status [20,21]. 

The questions in CHARLS were used to assess the health status.

Question (1): Have you been diagnosed with (conditions listed below, read one by one) by a doctor?

1. Hypertension
2. Dyslipidemia
3. Diabetes or high blood sugar
4. Cancer or malignant tumor (excluding minor skin cancers)
5. Chronic lung diseases, such as chronic bronchitis, emphysema (excluding tumors, or cancer)
6. Liver disease (except fatty liver, tumors, and cancer)
7. Heart attack, coronary heart disease, angina, congestive heart failure, or other heart problems
8. Stroke
9. Kidney disease (except for tumor or cancer)
10. Stomach or other digestive disease (except for tumor or cancer)
11. Emotional, nervous, or psychiatric problems
12. Memory-related disease
13. Arthritis or rheumatism
14. Asthma

Question 2: Would you say your health is excellent, very good, good, fair, or poor?

1. Excellent
2. Very good
3. Good
4. Fair
5. Poor

The outcome measures in our study were categorized as binary variables, and included the following: (1) been diagnosed with chronic disease or not; (2) good SAH (excellent, very good and good) and poor SAH (fair and poor). After age screening and data clean-up (i.e., excluding respondents of missing SAH (*n* = 37), missing chronic disease condition (*n* = 40), missing personal expenditure (*n* = 55)), 3460 respondents (259 older rural-to-urban migrant workers and 3201 older rural dwellers) were selected for this study.

### 2.3. Independent Variables

Numerous variables are available in CHARLS. Followed by prior empirical investigations [22,23,24,25,26,27], all possible variables that may produce migrants’ health were considered in our study. Four groups of variables were considered in this study. First, demographic characteristics were age group (50–54, 55–60, 61–65), gender (male, female), living arrangement (living with spouse, living without spouse present). Second, socioeconomic characteristics were education attainment (primary school and below, middle school, high school and above), medical scheme (yes (new cooperative medical scheme (NCMS), government medical scheme, medical aid, private medical scheme etc.), no), basic endowment scheme (yes, no), bath facility (hot water, no), monthly personal expenditure. The monthly personal expenditure was categorized into five income quantiles, with first quintile (bottom 20% expenditures households) represents the poorest expenditures quintile and fifth expenditures quintile represents the richest [28]. Third, geographic characteristics was the region (east, central, west). Fourth, health behavior variables were considered in this study were social activity, sleeping time at night (<4 h, 4–8 h, >8 h), smoking (yes, no), drinking (yes, no). Social activity was also measured by the question, “Have you done any of these activities in the last month?” Possible answers to this question were ten informal social interactions (e.g., interacted with friends, took part in a community-related organization). A social activity variable was created to count the number of social activities that represented the respondent’s participation during the last month. 

### 2.4. Coarsened Exact Matching Method

In line with Mark [29], migration for work is not randomly assigned, rather selective based upon personal circumstances. The covariate distributions of the data for those who migrated to work and those who did not is different. It is not possible to simply fit a regression model to analyze the health between older rural-to-urban migrant workers and older rural dwellers, as this would break the assumption of the model that there is no bias present in the data [29,30,31]. A crude comparison of the perception of the state of their health between older rural-to-urban migrant workers and older rural dwellers would also ignore the confounding factors. CEM can reduce the bias because older rural-to-urban migrant workers and older rural dwellers become (or very close to) identical in relation to individual characteristics [29,30]. Migration for work represents a change in status for individuals that can be tested [29]. By matching across multiple variables, the process controls for the effects of each variable allowing the analysis to focus more on the change in status (i.e., migration), which is the main difference between the two groups. CEM allows the analysis to focus on whether those who migrate for work have significantly different health when compared to those who did not. Compared with other matching methods, CEM has been found to yield estimates of the causal effect with the lowest variance and bias for any sample size [32]. Therefore, we employed CEM, which can perfect balance of distributions of the covariates and reduce model dependence between the comparison groups and thereby reducing the bias [17]. The basic algorithm of CEM mainly includes three procedures. Firstly, each variable is coarsened into groups and then performed the outcome estimation. In the second step, the algorithm of exact matching is employed to the coarsened data to determine the matches and to prune unmatched units. Finally, the coarsened data are discarded and the original (uncoarsened) values of the matched data are retained. Units in strata that contain at least one treated and one control unit are retained [32]. 

We have matched the two groups based on the employment status (be employed outside the country for six months or more in the past year), and “treatment” cases were older rural-to-urban migrant workers, and “non-treatment” controls were older rural dwellers. Variables are temporarily transformed into a series of (meaningful) categorical groups to get more accurate matches [31]. Age group, gender, living arrangement, educational attainment, social activity, sleeping time, smoking, and alcohol use were used in matching in our study. Matching weights generated by CEM were used to equalize the number of observations within comparison groups [32,33].

The multivariate imbalance measure L_1_ was used to check the global imbalance. L_1_ ranges from 0 (perfect global balance) to 1 (maximal imbalance), and larger value represents larger imbalance between comparison groups. A good matching performance would bring a substantial reduction in L_1_ [34]. The CEM method was modeled by using the “cem” command code in Stata.

### 2.5. Statistical Analysis

We provided descriptive statistics analysis that compared older rural-to-urban migrant workers with their rural counterparts (older rural dwellers). The differences were examined by chi-square test for categorical variables and the results were revealed in Table 1. After carrying out CEM, a logit model was fitted to find factors that were associated with the health status of older rural-to-urban migrant workers and older rural dwellers. Finally, we conducted Fairlie’s decomposition analysis to further explore the contributions of each dimension of health disparities. All statistical analysis was carried out by using STATA 15.0 (StataCorp LP., College Station, TX, USA). A *p*-value of less than 0.05 was considered statistically significant. Sampling weights, taking into account the selection probability of the individuals, were included in our analysis. 

### 2.6. Decomposition Method

Oaxaca-Blinder decomposition method has been exploited extensively to assess outcome differences in the discrimination and labor economics [35] to analyze the contribution of health disparities in different groups [36,37]. The main purpose of this decomposition model is to split health disparities into two components, explained and unexplained. The first part is explained by group differences in the distribution of observable variables and often regarded as “endowment” [38]; it is difficult to find any direct explanation for this unexplained gap from the decomposition analysis itself. The second part reflects the unobserved heterogeneity between the cohorts. Since our outcome variables were binary, our study utilized the non-linear decomposition methods proposed by Fairlie and Bartus, which was particularly suited to calculate disparities for binary variables as an extension of Blinder-Oaxaca decomposition technique to logit and probit Models [39,40]. 

Following Fairlie [39], the decomposition for a nonlinear equation, $Y=F(X\hat{\beta })$ can be written as:

(1) $${\bar{Y}}^{w}-{\bar{Y}}^{B}=\left[\sum_{i=1}^{N^{w}}\frac{F(X_{i}^{w}{\hat{\beta }}^{w})}{N^{w}}-\sum_{i=1}^{N^{B}}\frac{F(X_{i}^{B}{\hat{\beta }}^{w})}{N^{B}}\right]+\left[\sum_{i=1}^{N^{B}}\frac{F(X_{i}^{B}{\hat{\beta }}^{w})}{N^{B}}-\sum_{i=1}^{N^{B}}\frac{F(X_{i}^{B}{\hat{\beta }}^{B})}{N^{B}}\right]$$

where *N^j^* is the sample size for group *j*. In (1), the first term in brackets represents the part of the gap that is due to group differences in distributions of *X*, and the second term represents the part due to differences in the group processes determining levels of *Y*. The second term also captures the portion of the gap due to group differences in unmeasured or unobserved endowments. Similar to most of the previous studies [40,41] applying the decomposition technique, our study did not focus on this “unexplained” portion of the gap because of the difficulty in interpretation of results.

## 3. Result

### 3.1. Matching Performance

Table 1 demonstrated the descriptive statistics for the respondents. The L_1_ statistic dropped from 0.7243 before matching to close to zero after matching, which indicated good matching performances. 1517 respondents were successfully matched by the CEM method (215 older rural-to-urban migrant workers and 1302 older rural dwellers). As Table 1 illustrated, it was obvious that there were significant differences in many characteristics between the two groups before matching. The statistical difference between the two groups remarkably decreased in the matched populations, which also indicated good matching performances and thus different groups became more comparable. After matching, there were more older woman (60.93% vs. 72.13% respectively); more were in the age of 50–54 (40.93% vs. 42.59%); more lived with their spouses (90.70% vs. 95.03% respectively). Compared with older rural dwellers, older rural-to-urban migrant workers were more likely to have lower level of education, be less uninsured by medical and endowment schemes, have more social activities, have higher probability of sleep above 8 h, be higher probability of smoking and drink, be more in west region, be more in poorest and middle income quantiles. 

### 3.2. Description of Health Status 

To obtain a better sense of how the health status was distributed across the two matched groups, we draw the health distributions for these two groups separately in Table 2. It reported that older rural-to-urban migrant workers had better SAH than older rural dwellers; however, older rural-to-urban migrant workers were more likely to suffer from chronic diseases than their rural counterparts. 

### 3.3. Adjusted Associations between Health Status and its Determinants

Table 3 offered a variety of variables associated between health status and its determinants through logit models on SAH (1 = good SAH, 0 = bad SAH) and chronic disease condition (1= yes, 0= no). For older rural-to-urban migrant workers, no endowment scheme, living without spouse, sleeping time 4~8 h, middle region and poorer income quantiles were positive determinants of SAH; while 61~65 was a negative determinant of SAH. Meanwhile, for the older rural dwellers, west region was a positive determinant of SAH. For older rural-to-urban migrant workers, middle school was a positive determinant of chronic disease condition; for the older rural dwellers, no smoking and middle school were positive determinants of chronic disease condition; while woman, sleeping time 4~8 h and sleeping time >8 h were negative determinants of chronic disease condition. 

### 3.4. Decomposition Analysis

The core intention of our study was to parse out health disparities between older rural-to-urban migrant workers and older rural dwellers into observed and unobserved factors, once we accounted for all the explanatory variables in the model. We made mediation analysis, and found that only region actually had significant mediating effect in both SAH and chronic conditions. For the accuracy of the decomposition analysis, we removed region from the decomposition model. 10.44% of the health disparity of SAH was enlightened by the factors considered and 31.34% of the health disparity of chronic disease condition was also demonstrated in the current research. Weighted decomposition of the health disparity of the two groups was expressed in Table 4. Our findings confirmed that bath (−22.14%) was highly significant in clarification of differences in SAH; educational attainment (−14.40%), sleeping time (18.04%) and medical scheme (−11.19%) were highly significant in explanation of differences in chronic disease condition.

## 4. Discussion

To the best of our knowledge, it was the first large-scale comparative study in China to examine the health disparity specifically focused on the older rural-to-urban migrant workers (age 50 and above) in China, the country with the largest scale of internal migration. This paper provided new empirical evidences on the health outcomes between older rural-to-urban migrant workers and their rural counterparts. Matching helped reducing bias in the data, further allows for a more accurate and stronger analysis. 

Our results revealed that both older rural-to-urban migrant workers and older rural dwellers had high prevalence of chronic diseases, but they had better SAH, in consistent with rural-to-urban migrant workers of other ages [42]. These two health outcomes seemed apparently contradictory. It might be due to their ignorance of the health effects of chronic diseases. Specifically, they misunderstood chronic diseases as normal phenomena of the older, although they were more susceptible to the health disadvantage. Therefore, the government should pay special attention to popularizing health knowledge for the older and improving health literacy. Although many evidences [23,43,44] pointed that SAH was related to duration of chronic diseases, the severity of chronic diseases etc., we lacked accurate data to examine the relationship between SAH and chronic diseases of these older groups. Evidently, further studies were needed to take more details into account, to get a better understanding of the conflicting results among older rural-to-urban migrant workers and older rural dwellers. 

In comparison, the older rural-to-urban migrant workers were more likely to report better SAH, but a higher prevalence of chronic diseases than their rural counterparts. For SAH, older rural-to-urban migrant workers lived in cities with better health-care services and higher health infrastructures, providing a buffer against health risks, which were conducive to their SAH [45]. While older rural dwellers in rural areas were more likely to face limited accessibility to health care services and possible barriers to the diagnostic equipment or specialization to detect their health concerns. In line with prior studies of rural-to-urban migrant workers [8,46], older rural-to-urban migrant workers were more prone to chronic diseases, because of poor sanitation and working conditions, along with a desire to maximize earnings by working longer h, may also intensify the risk of developing chronic diseases. 

Our study also explored that the older rural-to-urban migrant workers in the west region accounted for a large proportion, just as National Bureau of Statistics reported, the absorption capacity of rural-to-urban migrant workers in the western region have gradually increased [1]. Unlike the related studies [47], an important finding was to note that older rural-to-urban migrant workers and older rural dwellers in the western region had better SAH than eastern region. It may be because the Chinese central government had promoted the acceleration of industrial transformation and up gradation in western cities—e.g., low taxation, national level fiscal transferring, policies encouraging people to migrate (including rural-to-urban migrant workers), thus brought high-quality and high-availability health services to the western cities [48]. Just as NBC [1] demonstrated, the rural-to-urban migrant workers in the east region mainly came from the west region and the middle region. Some older rural-to-urban migrant workers in east region worked far away from their home and families and they had faced many difficulties, such as high living expenses, large cultural and lifestyle differences, which deteriorated their health. Therefore, the question of whether older rural-to-urban migrant workers in western region were more vulnerable should be of interest to future study.

Many studies had studied the relationship between bathing water quality and health [49], and our study found that bath facility decreased SAH disparity of the two elderly groups. More older rural-to-urban migrant workers had hot water facilities than older rural dwellers. Thereby, the findings strongly suggest that we should measures to improving the bathing facility in rural.

As many studies had raised important questions about the significance of sleep deficiency as a driver of health disparities [50], our results corroborated that sleeping time at night decreased the disparity of chronic disease between older rural-to-urban migrant workers and older rural dwellers. Good sleep can occupy an important position in maintaining the body’s functions and promoting the physical health of the older [51,52], whose health are already more fragile than before. Therefore, to prevent sleep deficiency at night is an important consideration in health promotion, and reduction of healthy disparity between older rural-to-urban migrant workers and older rural dwellers.

Education was well known to narrow health disparities among the older [53], and our findings also illustrated that education decreased disparities of the chronic disease between older rural-to-urban migrant workers and older rural dwellers. It was partly due to the highly-educated older rural-to-urban migrant workers in cities can have better health-care services and can understand chronic disease prevention and control, so they can engage with regular check-ups; while highly-educated older rural dwellers with chronic health problems may face possible barriers to their health care utilization in rural. Therefore, awareness raising campaigns to test chronic diseases need be strengthened by health education programs, especially for the older rural dwellers without effective health management. 

Our regression analysis corroborated that medical scheme for the two older groups did not play an important role in promoting health [54]. But our study identified that medical scheme can decrease the health disparities in chronic disease between the two groups. Each city in China had its own medical scheme administration for rural-to-urban migrant workers, leading to different specific provisions from one city to another [55]. Considering the mobility of older rural-to-urban migrant workers, it’s difficult for them to settle their medical scheme accounts and claim reimbursement in different places. Studies suggested that improving medical scheme, upgrading a long-distance medical problem would be important for reducing the health disparity in chronic disease. Based on the findings, the Chinese government should consider how different options of medical scheme impact on efficiency for older rural-to-urban migrant workers when making policies.

Our results explained that there are no statistically significant differences in living expenses between these two groups. Despite migration may be financially rewarding, in the sense that it increased earning ability, older rural-to-urban migrant workers often did not use the money to promote their own well-being; they often sent money back to their place of origin for their left-behind family members, or they accumulate money for later, such as treatment and pension [56]. As a result, these detrimental factors to health may not be easily perceived at younger ages but accumulated at older ages. The older rural dwellers also frequently engaged in laborious agricultural work, which similarly will take a cumulative toll on health as they grow aged. 

A substantial share of the disparities still cannot be explained by the observed differences. But given that decomposition is conducted with common response scales, it is more likely that the inability to explain disparities in SAH and chronic disease condition is caused by data limitations, suggesting that we indeed capture differences in real health. 

We would also like to note the several limitations of our study. First, labor migration is a dynamic process, but the data in our study was predominantly cross-sectional and it is impossible to examine changes at different stages. Future longitudinal studies will need to track before and after labor migration and after returning to the rural. Second, CHARLS made the sample representativeness, and we selected all the older rural-to-urban migrants according to our criteria in the CHARLS data. The older rural-to-urban migrants were highly selected sample groups, and small sample sizes meant that some estimates lacked precision. It may still affect the representativeness of demographics distribution. The limited sample size for the older rural-to-urban migrant workers included did not allow for distinguishing older returning migrant workers and migrant workers who may be taking informal jobs or who may be staying with their adult children when they answered questionnaires. In addition, the missing information is not random. Pooled estimates were used to address this, however these may mask between groups heterogeneity. Third, we could not obtain certain variables (e.g., all chronic diseases, type of work, migration time) found to be associated with health status in previous studies in CHARLS questionnaire. There may be unknown confounding or mediating factors. Fourth, this study only discussed disparities between rural-to-urban migrants and their rural counterparts in China. However, urban living may have an overall impact on health (e.g., pollution, lifestyle etc.). We should also make a comparison between older rural-to-urban migrants and older urban dweller, to make the health disparities more comprehensive. Fifth, bias may exist in our study. Self-reported measures may lead to bias and analysis of complete cases may also lead to bias. Finally, CEM does not account for factors that affect assignment to treatment and outcome but that cannot be observed; therefore, any hidden bias due to latent variables may remain after matching [57]. 

## 5. Conclusions

This comparative study focusing on older rural-to-urban migrant workers and older non-migrant rural residents added to on-going and expanding literature in their health disparities. Compared with older rural dwellers, the healthy migrant phenomenon was observed among older rural-to-urban migrant workers on SAH, but not on chronic diseases. This study provided convincing evidence that priority should be given to improving chronic diseases for older rural-to-urban migrant workers. These findings also illustrated the main determinants of substantial health disparities between older rural-to-urban migrant workers and older rural dwellers—bath facility, sleeping time, medical scheme and education. Our study may help to offer evidence for future social policy and intervention strategies targeted to the drivers of health disparities in China’s long-standing division between rural and urban sectors.

## Figures and Tables

**Table 1:** Descriptive statistics of independent variables before and after coarsened exact matching (CEM).

Variable	Before Matching *N* (%)			After Matching *N* (%)			
	Older Rural-to-Urban Migrant Workers	Older Rural Dwellers	*p*-Value	Older Rural-to-Urban Migrant Workers	Older Rural Dwellers	*p*-Value *	*p*-Value #
Gender			0.195			0.089	1
Men †	110 (42.47)	1496 (46.74)		84 (39.07)	303 (27.87)		
Women	149 (57.53)	1705 (53.26)		131 (60.93)	784 (72.13)		
Age			0.588			0.849	0.4889
50–54 †	98 (38.58)	1236 (38.61)		88 (40.93)	463 (42.59)		
55–60	66 (25.98)	925 (28.90)		63 (29.30)	386 (35.51)		
61–65	90 (35.43)	1040 (32.49)		64 (29.77)	238 (21.90)		
Living arrangement			0.191			0.121	1
Live with spouse †	227 (87.64)	2699 (84.32)		195 (90.70)	1033 (95.03)		
Live without spouse	32 (12.36)	502 (15.68)		20 (9.30)	54 (4.97)		
Educational attainment			<0.05			0.085	1
Below primary school †	215 (84.65)	2542 (79.41)		79 (36.74)	317 (29.16)		
Primary school	31 (12.20)	461 (14.40)		70 (32.56)	406 (37.35)		
Middle school and above	8 (3.15)	198 (6.19)		66 (30.70)	364 (33.49)		
Medical scheme			0.352			0.466	0.3362
Yes †	219 (84.56)	2773 (86.63)		195 (90.70)	1002 (92.18)		
None	25 (9.65)	250 (7.81)		20 (9.30)	85 (7.82)		
Basic endowment scheme			< 0.05		< 0.05	0.059	0.1436
Yes †	169 (65.25)	2287 (71.45)		142 (66.05)	808 (74.33)		
None	90 (34.75)	914 (28.55)		73 (33.95)	279 (25.67)		
Social activity			< 0.01			0.073	1
None †	154 (59.46)	1576 (49.23)		130 (60.47)	764 (70.29)		
1	59 (22.78)	949 (29.65)		51 (23.72)	197 (18.12)		
≥2	46 (17.76)	676 (21.12)		34 (15.81)	126 (11.59)		
Sleeping time			0.774			0.630	1
≤4 †	38 (14.96)	419 (14.09)		26 (12.09)	121 (11.13)		
4–8	141 (55.51)	1859 (58.08)		130 (60.47)	746 (68.63)		
>8	75 (29.53)	923 (28.83)		59 (27.44)	220 (20.24)		
Smoke			< 0.05			0.072	1
Yes †	80 (31.50)	878 (27.43)		59 (27.44)	198 (18.22)		
No	174 (68.50)	2323 (72.57)		156 (72.56)	889 (81.78)		
Alcohol use			0.164			0.092	1
Yes †	104 (40.94)	1079 (33.71)		76 (35.35)	272 (25.02)		
No	150 (59.06)	2211 (66.29)		139 (64.65)	815 (74.98)		
Bath							
Yes †	108 (42.52)	1615 (50.45)		89 (41.40)	172 (15.93)		
No	146 (57.48)	1586 (49.55)		126 (58.60)	908 (84.07)		
Region			<0.001			<0.05	0.1673
East †	55 (21.24)	1223 (38.21)		47 (21.86)	406 (37.35)		
Middle	60 (23.17)	875 (27.34)		50 (23.26)	289 (26.59)		
West	144 (55.6)	1103 (34.46)		118 (54.88)	392 (36.06)		
Income quantiles			0.326			0.897	0.3826
Poorest †	71 (16.82)	2423 (22.90)		48 (22.33)	226 (20.79)		
Poorer	108 (25.59)	2268 (21.43)		42 (19.53)	214 (19.69)		
Middle	96 (22.75)	2136 (20.19)		50 (23.26)	224 (20.61)		
Richer	71 (16.82)	2164 (20.45)		30 (13.95)	236 (21.71)		
Richest	76 (18.01)	1590 (15.03)		45 (20.93)	187 (17.20)		
N	259	3201		215	1302		

Note: † Reference levels in the regressions; virtual variables for Chi-square test; * *p*-value indicated the actual *p*-values after matching; # *p*-value indicated the weight to be considered; *N* (%) were reported.

**Table 2:** Comparison of variables distribution in different health status between two matched groups.

Variable	Good SAH		Bad SAH		Suffering from Chronic Disease		Not Suffering from Chronic Disease	
	Older Rural-to-Urban Migrant Workers	Older Rural Dwellers	Older Rural-to-Urban Migrant Workers	Older Rural Dwellers	Older Rural-to-Urban Migrant Workers	Older Rural Dwellers	Older Rural-to-Urban Migrant Workers	Older Rural Dwellers
Gender								
Men †	77 (40.10)	7 (30.43)	244 (26.01)	59 (39.60)	65(38.92)	19(39.58)	216(26.93)	87(30.53)
Women	115 (59.90)	16 (69.57)	694(73.99)	90 (60.40)	102(61.08)	29(60.42)	586(73.07)	198(69.47)
Age								
50–54	79 (41.15)	9 (39.13)	404 (43.07)	59 (39.60)	76(45.51)	12(25.00)	358(44.64)	105(36.84)
55–60	62 (32.29)	1 (4.35)	327 (34.86)	59 (39.60)	43(25.75)	20(41.67)	278(34.66)	108(37.89)
61–65	51(26.56)	13 (56.52)	207 (22.07)	31 (20.81)	48(28.74)	16(33.33)	166(20.70)	72(25.26)
Living arrangement								
Live with spouse †	173 (90.10)	22(95.65)	893(95.20)	140 (93.96)	151(90.42)	44(91.67)	772(96.26)	261(91.58)
Live without spouse	19 (9.90)	1 (4.35)	45 (4.80)	9 (6.04)	16(9.58)	4(8.33)	30(3.74)	24(8.42)
Educational attainment								
Below primary school †	70 (36.46)	9(39.13)	271(28.89)	46 (30.87)	55(32.93)	24(50.00)	226(28.18)	91(31.93)
Primary school	64 (33.33)	6 (26.09)	353 (37.63)	53 (35.57)	52(31.14)	18(37.50)	298(37.16)	108(37.89)
Middle school and above	58 (30.21)	8 (34.78)	314 (33.48)	50 (33.56)	60(35.93)	6(12.50)	278(34.66)	86(30.18)
Medical scheme								
Yes †	175 (91.15)	20 (86.96)	961 (81.79)	141 (94.63)	148(88.62)	47(97.92)	728(90.77)	274(96.14)
None	17 (8.85)	3 (13.04)	77 (8.21)	8 (5.37)	19(11.38)	1(2.08)	74(9.32)	11(3.86)
Basic endowment scheme								
Yes †	123 (64.06)	19 (82.61)	699 (74.52)	109 (73.15)	105(62.87)	37(77.08)	586(73.07)	222(77.89)
None	69 (35.94)	4 (17.39)	239 (25.48)	40 (26.85)	62(37.13)	11(22.92)	216(26.93)	63(22.11)
Social activity								
None †	116(60.42)	14 (60.87)	666 (71.00)	98 (65.77)	105(62.87)	25(52.08)	560(69.83)	204(71058)
1	43 (22.40)	8 (34.78)	171 (18.23)	26 (17.45)	39(23.34)	12(25.00)	148(18.45)	49(17.19)
≥2	33 (17.19)	1 (4.35)	101 (10.77)	25 (16.78)	23(13.77)	11(22.92)	94(11.72)	32(11.23)
Sleeping time								
≤4	22 (11.46)	4 (17.39)	112(11.94)	9 (6.04)	24(14.37)	2(4.17)	110(13.72)	11(3.86)
4~8	120 (62.50)	10 (43.48)	640 (68.23)	106 (71.14)	98 (58.68)	32(66.67)	534(66.58)	212(74.39)
>8	50 (26.04)	9 (39.13)	186 (19.83)	34 (22.82)	45(25.95)	14(29.17)	158(19.70)	62(21.75)
Smoke								
Yes †	52 (27.08)	7 (30.43)	155 (16.52)	43 (28.86)	46 (27.54)	13(27.08)	127 (15.84)	71(254.91)
No	140 (72.92)	16 (69.57)	783 (83.48)	106 (71.14)	121 (72.46)	35(72.92)	675 (84.16)	214(75.09)
Alcohol use								
Yes †	70 (36.46)	6(26.09)	220(23.45)	52 (34.90)	58(34.73)	18(37.50)	195(24.31)	77(27.02)
No	122 (63.54)	17(73.91)	718(76.55)	97 (65.10)	109(65.27)	30(62.50)	607(75.69)	208(72.98)
Bath								
Yes †	77 (40.10)	12 (52.17)	414 (44.14)	86 (57.72)	69 (41.32)	20 (41.67)	349 (43.52)	151 (52.98)
No	115 (59.90)	11 (47.83)	524 (55.86)	63 (42.28)	98 (58.68)	28 (58.33)	453 (56.48)	134 (47.02)
Region								
East †	38 (19.79)	9 (39.13)	331 (35.29)	75 (50.34)	34(20.36)	13(27.08)	278(34.66)	128(44.91)
Middle	44 (22.92)	6 (26.09)	251(26.76)	38 (25.50)	33(19.76)	17(35.42)	225(28.05)	64(22.46)
West	110 (57.29)	8 (34.78)	356 (37.95)	365 (24.16)	100(59.88)	18(37.50)	299(37.28)	93(32.63)
Income quantiles								
Poorest †	41 (21.35)	7 (30.43)	202 (21.54)	24 (16.11)	37(22.16)	11(22.92)	168(20.95)	58(20.35)
Poorer	41 (21.35)	1 (4.35)	188(20.04)	26 (17.45)	34(20.36)	8(16.67)	155(19.33)	59(20.70)
Middle	41 (21.35)	9 (39.13)	185 (19.72)	39 (26.17)	36(21.56)	14(29.17)	148(18.45)	76(26.67)
Richer	27 (14.06)	3 (13.04)	204(21.75)	32 (21.48)	29(17.37)	1(2.08)	182(22.69)	54(18.95)
Richest	41 (21.88)	3 (13.04)	159 (16.95)	28 (18.79)	31(18.56)	14(29.17)	149(18.580)	38(13.33)
N	192(89.30)	23 (10.70)	938 (86.29)	149 (13.71)	167(76.77)	48(22.33)	822(73.78)	285(26.22)

Note: † Reference levels in the regressions; *N* (%) were reported.

**Table 3:** Matched multivariate regression results of older rural-to-urban migrant workers vs. older rural dwellers.

Variable	SAH						Chronic Disease Condition					
	Older Rural-to-Urban Migrant Workers			Older Rural Dwellers			Older Rural-to-Urban Migrant Workers			older Rural Dwellers		
	Coefficient	[95% CI]		Coefficient	[95% CI]		Coefficient	[95% CI]		Coefficient	[95% CI]	
Gender	−1.5718	−4.4291	1.2856	0.0378	−0.6869	0.7625	−0.2126	−1.4023	0.9772	−0.8626 ***	−1.5119	−0.2133
Age												
55–60	1.6919	−0.8663	4.2501	−0.2398	−0.6521	0.1724	−0.6197	−1.6093	0.3700	−0.2805	−0.6110	0.0501
61–65	−1.8364 *	−3.6417	−0.0310	−0.1102	−0.6367	0.4163	−0.3953	−1.3944	0.6039	−0.2352	−0.6386	0.1682
Living arrangement	3.9501 *	0.8196	7.0807	−0.0812	−0.8790	0.7165	0.5437	−0.8025	1.8900	−0.5560	−1.1678	0.0559
Educational attainment												
Primary school	−0.3456	−2.2686	1.5774	0.0548	−0.3984	0.5081	−0.0809	−0.9503	0.7886	0.0270	−0.3279	0.3820
Middle school and above	−0.5405	−2.5374	1.4563	−0.0174	−0.4987	0.4638	1.2849 **	0.1313	2.4385	0.2530	−0.1290	0.6351
Medical scheme	−0.5099	−3.2417	2.2219	0.1897	−0.5964	0.9757	1.6634	−0.5694	3.8963	0.5960	−0.0816	1.2736
endowment scheme	2.4554 *	0.2016	4.7091	0.0531	−0.3595	0.4658	0.9223	−0.0404	1.8849	0.2240	−0.1193	0.5674
Social activity												
1	−1.1014	−2.8714	0.6686	0.1575	−0.3427	0.6577	−0.4311	−1.4568	0.5946	0.2068	−0.1819	0.5954
≥2	2.7588	−0.3220	5.8396	−0.2825	−0.8409	0.2758	−0.7236	−1.7992	0.3521	0.3164	−0.1721	0.8050
Sleeping time												
4~8	2.2319 *	0.0519	4.4119	−0.3607	−1.1074	0.3860	−1.1958	−2.8496	0.4581	−1.3422 ***	−2.0044	−0.6800
>8	1.2174	−1.0711	3.5059	−0.5215	−1.3348	0.2917	−0.9221	−2.6711	0.8269	−1.3115 ***	−2.0259	−0.5971
Alcohol use	0.2774	−1.8238	2.3786	0.1804	−0.1370	0.4978	0.6628	−0.3256	1.6511	0.0184	−0.2504	0.2872
Smoke	2.6763	−0.1549	5.5075	0.3907	−0.2965	1.0779	−0.1906	−1.4793	1.0980	1.2873 **	0.6626	1.9119
Bath	0.6029	−0.9571	2.1629	0.5189	0.1279	0.9099	−0.3518	−1.2942	0.5905	0.3081 *	0.0017	0.6146
Region												
Middle	2.9654 **	−0.5666	5.3642	0.3566	−0.0941	0.8074	−0.5750	−1.7166	0.5666	0.4815 **	0.1059	0.8571
West	3.1471 ***	1.0595	5.2347	0.6995 **	0.2494	1.1497	0.4132	−0.6000	1.4264	0.3377	−0.0026	0.6780
Income quantiles												
Poorer	5.6175 **	1.5209	9.7140	0.0637	−0.5587	0.6861	0.3611	−0.9562	1.6783	0.1077	−0.3444	0.5599
Middle	0.3727	−1.4043	2.1497	−0.4378	−1.0112	0.1356	−0.6025	−1.7702	0.5651	−0.2560	−0.6916	0.1797
Richer	1.7620	−1.2203	4.7444	−0.1380	−0.7218	0.4458	2.2093	−0.1301	4.5486	0.3146	−0.1331	0.7623
Richest	2.2512	−0.1742	4.6766	−0.2623	−0.8752	0.3505	−0.3599	−1.5185	0.7987	0.3789	−0.1160	0.8738

Note: 95% Coefficient Interval: 95%CI; * *p* < 0.05, ** *p* < 0.01, *** *p* < 0.001; all predictors entered the multivariate regression simultaneously.

**Table 4:** Fairlie’s decomposition of health disparity between matched older rural-to-urban migrant workers and older rural dwellers.

Terms of Decomposition	SAH			Chronic Disease Condition		
Total gap (%)	−0.0354			−0.0299		
Explained (%)	−0.0036 (10.44%)			−0.0093 (31.34%)		
**Explained**						
**Contribution to difference**	**Contribution (%)**	**95%CI**		**Contribution (%)**	**95%CI**	
Gender	7.74	−0.0056	0.0107	−13.44	−0.0052	0.0107
Age	−12.48	−0.0027	0.0017	−0.29	−0.0019	0.0025
Living arrangement	7.17	−0.0026	0.0025	−0.23	−0.0022	0.0025
Educational attainment	10.44	−0.0018	0.0029	−14.40 *	0.0004	0.0087
medical scheme	12.57	−0.0012	0.0112	−11.19 *	0.0002	0.0048
Endowment scheme	−7.21	−0.0041	0.0041	13.88	−0.0083	0.0004
Social activity	5.75	−0.0027	0.0045	−1.28	−0.0027	0.0040
Sleeping time	10.55	−0.0014	0.0075	18.04 **	−0.0097	0.0001
Alcohol use	−13.80	−0.0090	0.0050	−10.88	−0.0032	0.0089
Smoke	18.46	−0.0021	0.0063	13.99	−0.0134	0.0035
Bath	−22.14 *	−0.0102	0.0012	5.04	−0.0047	0.0019
Quantiles	−6.61	−0.0097	0.0075	13.05	−0.0154	0.0075

Note: a logit regression model on a pooled sample was run; 95% Coefficient Interval: 95%CI; * *p* < 0.05, ** *p* < 0.01, *** *p* < 0.001.

## Abbreviations

NBC	National Bureau of Statistics
CHARLS	China Health and Retirement Longitudinal Study
CEM	coarsened exact matching method
SAH	self-assessed of health status
Hukou	Chinese household registration system
NCMS	New cooperative medical scheme
95%CI	95% Coefficient Interval
